# Advancing Crop Resilience Through High-Throughput Phenotyping for Crop Improvement in the Face of Climate Change

**DOI:** 10.3390/plants14060907

**Published:** 2025-03-14

**Authors:** Hoa Thi Nguyen, Md Arifur Rahman Khan, Thuong Thi Nguyen, Nhi Thi Pham, Thu Thi Bich Nguyen, Touhidur Rahman Anik, Mai Dao Nguyen, Mao Li, Kien Huu Nguyen, Uttam Kumar Ghosh, Lam-Son Phan Tran, Chien Van Ha

**Affiliations:** 1 National Key Lab for Plant Biotechnology, Agricultural Genetics Institute, Ha Noi 100000, Vietnam; hoanguyentb.agi@gmail.com; 2 Institute of Genomics for Crop Abiotic Stress Tolerance, Department of Plant and Soil Science, Texas Tech University, Lubbock, TX 79409, USA; nhi.pham@ttu.edu (N.T.P.); tanik@ttu.edu (T.R.A.); maidao@ttu.edu (M.D.N.); 3 Department of Agronomy, Bangabandhu Sheikh Mujibur Rahman Agricultural University, Gazipur 1706, Bangladesh; uttam@bsmrau.edu.bd; 4 Vietnam National University of Agriculture, Ha Noi 100000, Vietnam; ntthuong355@gmail.com; 5 FPT University, Quy Nhon 590000, Vietnam; thuntb3@fe.edu.vn; 6 Donald Danforth Plant Science Center, Saint Louis, MO 63132, USA; mli@danforthcenter.org; 7 Department of Genetics Engineering, Agricultural Genetics Institute, Ha Noi 100000, Vietnam; kienbio280888@gmail.com

**Keywords:** high-throughput phenotyping, hyperspectral imaging, machine learning, plant stress tolerance, unmanned aerial vehicles

## Abstract

Climate change intensifies biotic and abiotic stresses, threatening global crop productivity. High-throughput phenotyping (HTP) technologies provide a non-destructive approach to monitor plant responses to environmental stresses, offering new opportunities for both crop stress resilience and breeding research. Innovations, such as hyperspectral imaging, unmanned aerial vehicles, and machine learning, enhance our ability to assess plant traits under various environmental stresses, including drought, salinity, extreme temperatures, and pest and disease infestations. These tools facilitate the identification of stress-tolerant genotypes within large segregating populations, improving selection efficiency for breeding programs. HTP can also play a vital role by accelerating genetic gain through precise trait evaluation for hybridization and genetic enhancement. However, challenges such as data standardization, phenotyping data management, high costs of HTP equipment, and the complexity of linking phenotypic observations to genetic improvements limit its broader application. Additionally, environmental variability and genotype-by-environment interactions complicate reliable trait selection. Despite these challenges, advancements in robotics, artificial intelligence, and automation are improving the precision and scalability of phenotypic data analyses. This review critically examines the dual role of HTP in assessment of plant stress tolerance and crop performance, highlighting both its transformative potential and existing limitations. By addressing key challenges and leveraging technological advancements, HTP can significantly enhance genetic research, including trait discovery, parental selection, and hybridization scheme optimization. While current methodologies still face constraints in fully translating phenotypic insights into practical breeding applications, continuous innovation in high-throughput precision phenotyping holds promise for revolutionizing crop resilience and ensuring sustainable agricultural production in a changing climate.

## 1. Introduction

High-throughput phenotyping (HTP) is an automated technique used for efficiently generating data on specific traits and attributes of large plant populations [1,2,3]. This approach integrates advanced imaging systems, sensors, and automated platforms to enable precise, rapid, and non-destructive trait measurements, facilitating comprehensive plant trait analyses. HTP platforms primarily utilize spectroscopy-based methods to assess phenotypic traits, while high-performance computing systems process large datasets to evaluate morphological, physiological, and biochemical responses, growth dynamics, and gas exchange rates in plants [4]. By allowing the simultaneous observation of plants at various growth stages under various environmental conditions and the collection of multiple trait datasets across large populations, HTP enhances the efficiency and accuracy of plant phenotyping [5]. However, when using HTP platforms to evaluate plant performance under field stress conditions, a major challenge is maintaining consistent uniform stress exposure to ensure experiment reproducibility across years [4,5]. The effectiveness of plant stress-responsive phenotyping is often influenced by variations in vegetative development and genotype-by-environment (G × E) interactions, which can confound the accurate assessment of plant stress responses. To address these challenges, controlled environments, such as growth chambers, greenhouses, and rainout shelters, are essential for ensuring comparability across genotypes. Experimental designs should also account for variations in stress intensity due to plant growth differences, enhancing the reliability of phenotypic assessments. Advanced HTP approaches, such as time-series imaging and high-throughput physiological measurements, further improve stress response tracking, while machine learning models aid in distinguishing true genetic differences from environmental variability, thereby increasing the accuracy of genotype evaluation under stress conditions [5,6]. Furthermore, HTP can play a vital role in integrating these technologies into crop breeding programs. Modern HTP platforms leverage high-resolution digital imaging, three-dimensional (3D) point cloud data, hyperspectral and multispectral imaging, and thermal imaging to enhance the phenotypic assessments of segregating plant populations under breeding programs. These systems also incorporate data modeling and geometric and radiometric correction to improve measurement precision [6]. As digital phenotyping technologies continue to evolve, their adoption is expected to expand globally, facilitating advancements in crop research and breeding efforts [6]. Despite their transformative potential, the adoption of HTP technologies remains limited, particularly in resource-constrained regions where traditional phenotyping methods continue to be widely utilized. Historically, plant phenotyping relied on manual, labor-intensive, and time-consuming techniques that often made the process expensive and inefficient [7,8,9,10,11,12,13]. Rather than replacing conventional approaches, HTP may serve as a complementary tool that enhances the efficiency and scalability of phenotyping. Usually, crop traits are quantified using destructive sampling methods, requiring multiple interventions throughout the growing season, which not only increases the risk of human errors but also hinders continuous monitoring [14,15,16,17]. For instance, biomass and grain yield assessments typically involve harvesting plant tissues at specific developmental stages, limiting the ability to track plant responses over time. In contrast, non-destructive HTP approaches allow for real-time, high-throughput data collection across diverse environmental conditions, significantly enhancing the precision and efficiency of traits measurement [18,19,20].

Climate change-driven factors are posing substantial challenges to modern agriculture [21,22,23,24]. The economic impact of these environmental pressures is projected to be significant, with estimates suggesting a potential decline of up to 18% in global gross domestic product by 2050, and even greater losses anticipated by the end of the century if effective mitigation strategies are not implemented [21,25]. Among the primary contributors to reduced agricultural productivity and economic losses are plant diseases, which can lead to yield reductions of 20–30%, depending on crop type and severity of infection [26,27]. Plant stress arises when crops encounter unfavorable environmental conditions, which can be broadly classified into abiotic and biotic stressors. Abiotic factors include drought, salinity, extreme temperatures, light limitations, nutrient deficiencies, heavy metal stress, wind stress, etc., while biotic stressors encompass insect infestations and pathogen attacks [28,29]. The emergence and recurrence of plant diseases are governed by intricate interactions among host plants, pathogens, and environmental conditions, making plant health management increasingly complex in the face of erratic climate patterns [29,30,31]. In responses to these stresses, plants activate a range of defense mechanisms involving morphological, physiological, biochemical, and molecular adaptations to mitigate stress-induced damage and enhance survival [32,33,34]. For instance, drought reduces water availability, limits net photosynthesis, and often shifts resource allocation towards root development at the expense of shoot growth to improve water and nutrient absorption. Similarly, salinity disrupts water uptake, induces ion imbalance, and damages cellular functions, typically leading to reduced shoot growth, while promoting root expansion as an adaptive response [35]. Heat stress can denature proteins and impair photosynthetic efficiency, while cold stress alters membrane fluidity and disrupts metabolic pathways critical for plant development [36,37,38]. The phenotyping of plant stress research the facilitates early detection and characterization of crop responses to abiotic and biotic stressors [20,32,33], providing valuable insights for breeding stress-resilient genotypes. Moreover, integrating HTP with genomic, ecological, and agronomic research has positioned modern phenotyping as a cornerstone of crop improvement programs, accelerating the development of stress resilient cultivars and sustainable agricultural practices [7,13]. The challenges of climate change highlight the urgent need to develop resilient crop varieties using advanced technologies to safeguard global food security.

This review provides a comprehensive synthesis of advancements in plant HTP technologies and their transformative role in plant stress research and crop improvement. The discussion is structured into (i) exploring cutting-edge HTP innovations, including non-invasive imaging techniques, spectroscopy, and automated sensor platforms, highlighting their role in enhancing the accuracy and efficiency of trait assessments; (ii) examining how HTP technologies facilitate the analyses of monogenic, oligogenic, and multigenic traits, enabling genomic predictions and marker-assisted selection strategies to enhance parental material selection and hybrid development; and (iii) addressing critical challenges in HTP research, including the management of large datasets, accessibility of HTP technologies, and scalability of HTP platforms. Additionally, this review underscores the growing importance of HTP technologies in plant stress research and their potential to drive innovations in crop breeding and sustainable agricultural management.

## 2. HTP in Plant Stress Response and Crop Improvement

**Figure 1:**
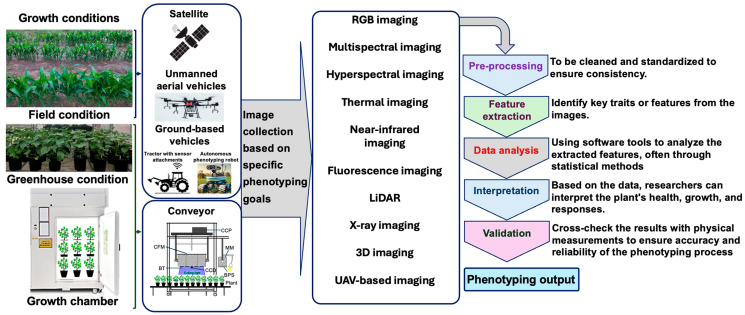
A schematic illustration of high-throughput phenotyping, highlighting multiple phenotyping approaches to enable efficient and comprehensive analysis. The figure emphasizes the synergy among different techniques including imaging, sensor-based systems, and automated data collection to enhance the precision and scalability of phenotypic assessment. BPS, band-pass filter; BT, blue light-emitting diode tube; CFM, chlorophyll fluorescence module; CCD, charge-coupled device camera; CCP, circuit control panel; LiDAR, light detection and ranging; MM, multispectral module; RGB, red–green–blue; UAV, unmanned aerial vehicle; 3D, three-dimensional.

HTP enables the rapid and accurate evaluation of plant phenotypic characteristics such as growth stage, stress adaptation, disease resistance, pest susceptibility, physiology, nutrition, and yield [39]. Integrating HTP, trait-based data analysis, and artificial intelligence (AI)-driven automation has improved our understanding of plant–environment interactions [36,40,41,42,43]. One of the key advantages of HTP is its ability to efficiently measure plant traits across large populations under diverse environmental conditions [39,43,44]. HTP technologies allow researchers to collect data at various scales, ranging from whole plants to cellular and molecular levels, with unprecedented efficiency [39,45,46]. As a result, HTP has revolutionized plant breeding programs by enabling large-scale phenotyping, accelerating variety improvement, and refining our understanding of the dynamic interactions between plant genetics and environments [39]. Figure 1 illustrates a schematic representation of HTP, highlighting various phenotyping strategies and their contributions to advancing research in plant stress responses and variety improvement.

A fundamental technology in HTP is remote sensing, which employs aerial and satellite-based systems to capture detailed phenotypic data across large agricultural landscapes. The effectiveness of satellite-based remote sensing largely depends on image resolution, which can be influenced by environmental variability [44]. Remote sensing techniques provide a powerful tool for monitoring crop growth and development, detecting stress responses, identifying nutrient imbalance, and diagnosing disease outbreaks at early stage [39]. HTP platforms integrated with diverse imaging devices and sensors have been adopted to assess key agronomic traits, including vegetation indices (VIs) [47,48,49,50]. VIs are particularly valuable in HTP programs, offering non-destructive insights into plant phenotype, structure, stress levels, and biomass accumulation [51]. VIs are computed using multiple spectral bands to extract vegetation-related features and assess plant cover, vigor, and biomass on a per-pixel basis [52]. A range of spectral indices exists to evaluate different physiological and morphological characteristics from remote sensing imagery [53,54]. The selection of appropriate indices is vital for optimizing their application in specific phenotyping studies [55,56]. Near-infrared (NIR) and infrared (IR) wavelengths are commonly used to assess plant responses to biotic and abiotic stresses, as healthy vegetation typically reflects higher levels of IR light compared with stressed plants [57,58,59]. Table 1 lists several widely used VIs for phenotyping to assess plant stress responses.

### 2.1. UAV-Based HTP

Unmanned aerial vehicles (UAVs) have emerged as a powerful tool in HTP, revolutionizing plant breeding by generating extensive datasets on crop canopy spectral reflectance. Datasets serve as UAV-HTP-derived traits, providing integrated measurements of canopy architecture and photosynthetic performance based on light reflectance from crop canopies [68,69,70,71]. Among remote sensing platforms, UAV imaging is considered the most practical and cost-effective approach for large-scale HTP studies under field conditions. During flight, UAVs capture multiple images that cover specific portions of a field. Images are then stitched together to form an orthomosaic, georeferenced composite image that represents the entire study area. Several software programs including Pix4D (a photogrammetry and drone mapping software) and quantum geographic information system (QGIS) and OpenDroneMap are commonly used for orthomosaic generation and analysis [72]. Compared with satellites, UAVs fly at lower altitudes, enabling them to capture images with significantly higher spatial resolution often in the range of a few centimeters per pixel, as opposed to the meter-scale resolution of satellite imagery. Additionally, UAVs possess greater payload capacities, allowing them to carry sophisticated sensors that are typically mounted on satellite platforms [73]. While UAVs have a more limited coverage area due to constraints in flight duration and battery capacity, they offer unparalleled advantages in spatial resolution, data accuracy, and operational flexibility. UAVs characteristics make an ideal choice for targeted phenotyping applications in plant stress research and crop improvement programs [74,75]. Machine learning models offer a promising and efficient approach for phenotyping the stay-green trait in sorghum (*Sorghum bicolor*), enhancing breeding efforts but requiring further validation across diverse environments and genetic materials [75]. On the other hand, satellite-based platforms are subsequently analyzed using spectral indices to assess plant traits and stress responses [76,77,78]. Hyperspectral imaging enables the detection of a broad spectrum of spectral signatures from plants, providing valuable insights into their biochemical and physiological properties. In addition, 3D imaging facilitates the reconstruction of plant structures, allowing for precise measurements of structural traits such as leaf surface area, leaf orientation, and plant stature. The ability to collect high-resolution phenotypic data at a large scale is particularly crucial in addressing challenges posed by climate change, as it enhances the accuracy of crop productivity predictions and resilience assessments [6].

### 2.2. Non-UAV-Based HTP

Advancements in robotics and automation are also vital for enhancing non-UAV-based HTP, facilitating precise and efficient phenotyping assessments in both controlled greenhouse environments and large-scale field settings [79,80,81]. In greenhouse conditions, where environmental factors can be optimized, non-UAV-based HTP technologies offer reliable monitoring, enabling extensive data collection. Automated systems, including robotic arms, conveyors, and high-definition cameras, can autonomously perform tasks, such as irrigation, fertilization, and imaging, reducing the need for human intervention [77,82,83,84]. Although drones depend on solar radiation for imaging, robotic systems can operate independently of weather conditions by using artificial lighting for plant scenes [85,86]. Furthermore, the integration of computer vision with robotics plays a significant role in automating manufacturing processes and phenotyping tasks [87]. These approaches have shown great potential in facilitating real-time data collection and analysis, enabling large-scale crop screening. As a result, non-UAV-based HTP platforms accelerate crop breeding programs by improving trait selection and enhancing the efficiency of phenotypic assessments. Additionally, automation not only reduces labor costs but also ensures greater accuracy and consistency in phenotypic evaluations, making it an essential tool in crop improvement research [88].

### 2.3. Integration of Machine Learning and Deep Learning Potential with AI in HTP

The integration of machine learning, particularly deep learning approaches, has further revolutionized HTP by enabling the extraction of meaningful insights from complex datasets [89]. Machine learning-driven phenotyping plays a pivotal role in identifying genotype–phenotype relationships that were previously difficult to be elucidated. Among the deep learning techniques, convolutional neural networks (CNNs) have demonstrated high efficacy in image-based phenotyping, automating the measurement of plant traits, such as growth rates and stress responses [37]. HTP workflows incorporating deep learning technologies typically involve multiple stages, beginning with data collection, labeling, and curation to ensure high-quality input for analysis [90]. Data can be acquired from various sensors, including red–green–blue (RGB), hyperspectral, multispectral, near-infrared (NIR), fluorescence, and light detection and ranging (LiDAR) [91]. Following data acquisition, model selection and optimization strategies are applied to improve computational efficiency and accelerate model training. The development of deep learning models follows an iterative process, allowing for continuous refinement and task-specific adaptation [92]. Compared with conventional phenotyping methods, deep learning-based applications significantly streamline HTP workflows, making large-scale phenotypic analysis more accessible and efficient [76]. Ultimately, the HTP approach enables the transformation of large volumes of phenotypic data into actionable insights, driving advancements in the field of crop variety improvement and plant stress research [83]. Research on managing biotic and abiotic stressors in field crops using a variety of machine learning and deep learning techniques is summarized in Table 2.

While HTP technologies have demonstrated significant potential in plant research, many existing studies, including those referenced in Table 2, remain at the validation or calibration stage in real-world experiments. HTP studies primarily focus on refining measurement accuracy, optimizing analytical pipelines, and enhancing the reproducibility of phenotypic assessments under both controlled and field conditions. Despite this, the rapid advancements in sensor technology, imaging techniques, and data processing capabilities suggest that HTP approaches will soon transition from being used primarily for experimental validation to become a routine component of crop breeding programs. The integration of HTP in large-scale breeding efforts is expected to revolutionize plant selection by enabling precise, non-destructive, and high-throughput assessments of key agronomic traits. Images obtained from high-throughput imaging systems can also be processed and analyzed using AI algorithms, enabling the identification of key patterns and traits within the plant phenotypic data. The ability of AI to assess plant growth factors, distinguish leaf morphologies, and detect early signs of disease symptoms makes it a very useful tool in plant phenotyping [106]. Within the framework of HTP, AI algorithms utilize a range of data types, including environmental conditions, genomic information, and imaging data, to evaluate and better understand plant traits [39]. By integrating diverse datasets, AI methodologies can uncover complex relationships between the environment, phenotype, and genetics, facilitating deeper insights into plant development. Furthermore, AI-driven predictive models that correlate phenotypic data with other factors such as genotype and environmental conditions advance our capacity to predict and optimize plant growth and resilience in various agricultural contexts [107].

### 2.4. Integration of HTP-Derived Traits with Genomic

HTP platforms offer potential in connecting genotypes to phenotypes by enhancing data collection capabilities (beyond visual range, repeated measurements, and big data processing), boosting measurement precision, and reducing the time needed to assess field populations on a large scale [108]. HTP can be effectively implemented across multiple growth stages and diverse environmental conditions, significantly enhancing phenotypic datasets and increasing the accuracy of accession selection from segregating population in plant breeding programs [109,110]. The combination of HTP with advanced genomic techniques, such as genome-wide association studies (GWAS) and quantitative trait locus (QTL) mapping, has substantially advanced the understanding of the genetic architecture underlying complex quantitative traits in crops [5]. The integration of HTP-derived traits with genomic facilitates the accelerated development of high-yielding varieties with improved resilience to climate change-induced stresses [71]. Research has been dedicated to elucidating the genetic mechanisms of stress tolerance, including phosphorus deficiency in rapeseed (*Brassica napus*) [111], drought responses in barley (*Hordeum vulgare*) [109], salinity tolerance in rice (*Oryza sativa*) [112], and drought tolerance in both rice and maize (*Zea mays*) [72,113]. Advancements in plant phenomics and genomics have provided valuable insights into the genetic basis of stress tolerance, enabling more efficient plant breeding strategies for crop improvement.

## 3. Limitations and Challenges of HTP in Agricultural Applications

Advanced HTP methods in plant stress and crop improvement research face several limitations that hinder their efficiency in agricultural applications.

Data storage and real-time processing: A key obstacle for large-scale applications in precision agriculture lies in data storage and immediate data processing. Hyperspectral imaging produces large-scale volumes of data, which represents a major challenge presenting major contests for efficient data storage and analyses. Each hyperspectral image can encompass hundreds to thousands of spectral bands, resulting in large file sizes that demand substantial storage capacity [114]. The demand for the swift and efficient processing of these huge datasets to deliver immediate data-driven insights necessitates extensive computational capacity and optimized logic sequences. Additionally, handling and preserving the accuracy of these large datasets over time may pose further challenges, especially in isolated or resource-constrained agricultural areas [114].

Cost and accessibility: Another limitation for large-scale implementation is the cost and accessibility of advanced HTP technologies in individual and resource-poor agricultural areas. The substantial upfront cost of hyperspectral imaging equipment continues to be a major obstacle to extensive adoption, especially for small- to mid-scale agricultural enterprises. However, hyperspectral approaches have proven useful for advancing multispectral investigations by identifying the most relevant reflectance bands, which is essential for the further design of multispectral cameras and their applications.

Field conditions and environmental variability: Agricultural environments are inherently diverse, with varying soil types and unpredictable meteorological patterns, all of which can affect the precision of spectral reflectance measurements. For instance, fluctuations in solar intensity and angle may result in irregular spectral sensor measurements. Soil hydration, texture, and arrangement also affect plant spectral traits, making it difficult to ensure consistent data integrity throughout fields.

Incorporation with existing farming practices: The effortless incorporation of hyperspectral imaging techniques into prevailing agricultural practices can be challenging and possibly destabilizing. Agricultural researchers or farmers familiar with conventional practices may find that the introduction of new technologies requires adjustments in workflows and training. Integrating new farming technologies with digital phenotyping tools must be seamless, ensuring efficiency without disrupting agricultural processes.

Standardization and regulatory issues: A lack of standardized protocols is a significant barrier to the seamless integration and comparison of results across various studies and platforms. Without a common framework, researchers face challenges in replicating and validating findings, which hinders scientific progress and collaboration. Additionally, researchers and operators must follow regulations when using drones for agricultural research. In the United States, the Federal Aviation Administration (FAA)’s Part 107 rules require remote pilot certification or special approval for legally flying drones over field trials [115]. Furthermore, FAA rules also require UAV operator registration and pilot training. Globally, UAV operations must comply with diverse national and regional regulations, further complicating the widespread adoption of drone technology in agricultural practice [116]. By overcoming traditional phenotyping limitations, such as labor-intensive data collection and environmental variability, HTP can facilitate the more efficient screening of stress-tolerant genotypes. Future research should focus on standardizing data acquisition protocols, improving the cost-effectiveness of HTP platforms, and addressing challenges related to large-scale deployment in breeding trials. As HTP technologies continue to evolve, their incorporation into breeding pipelines will enhance selection efficiency and accelerate genetic gains in crop improvement programs.

## 4. Conclusions

As climate change intensifies and the need for resilient crops becomes more urgent, advanced phenotyping technologies are set to play a pivotal role in modern agriculture. Overcoming current limitations through interdisciplinary collaboration and investment in scalable, accessible solutions will be essential to fully harness the potential of HTP in ensuring a sustainable agriculture. The continued development of HTP tools holds a great promise for accelerating advancements in plant stress research, crop breeding, and phenotyping technologies. However, deployment in precision phenotyping may differ significantly in field applications, posing challenges for widespread implementation across diverse agricultural settings. To enable seamless integration, the design of high-performance, cost-effective HTP solutions must remain a priority, ensuring reliable, automated data collection from diverse environments. One of the most pressing challenges for future HTP applications is managing the vast amounts of sensor-generated data and imagery produced by multifunctional phenotyping platforms. The complexity of data handling, retention, and interpretation will continue to grow, influenced by sensor resolution and the number of variables measured. To facilitate broader adoption of HTP in crop improvement programs, the development of cost-effective and accessible data analysis infrastructure will be critical, enabling researchers and breeders to efficiently extract meaningful insights.

## Figures and Tables

**Table 1:** Commonly employed vegetation indices for assessing plant stress responses.

Indices and Abbreviations	Formulae	Wavelengths	Applications	References
Normalized difference vegetation index (NDVI)	(MIR − NIR)/(MIR + NIR)	800, 10, 10, 1300:3000, variables: MIR = [1300:3000], NIR = [800;10;10] mit MIR = 1300 bis 3000 nm	Plant condition monitoring; measuring plant stress responses	[60]
Normalized green–red difference index (NGRDI)	(Rg − Rr)/(Rg + Rr)	490:570, 640:760	Biomass measurements	[60]
Visible atmospherically resistant indices (VARI700)	(Rg − Rr)/(Rg + Rr − Rb)	470:490, 660:680, 700	Accentuating plant life in images	[61]
Green leaf index (GLI)	(2 × Rg − Rr − Rb)/(2 × Rg + Rr + Rb)	420:480, 490:570, 640:760	Differentiating plant cover from exposed soil	[62]
Green normalized difference vegetation index (GNDVI)	(Rn − Rg)/(Rn + Rg)	540:570, 780:1400	Leaf chlorophyll measurements	[63]
Triangular greenness index (TGI)	Rg − 0.392 × Rr − 0.612 × Rb	475:485, 480, 545:555, 550, 665:675, 670		[64]
MERIS (medium resolution imaging spectrometer) terrestrial chlorophyll index (MTCI)	(R750 − R710)/(R710 − R680)	681, 709, 754		[65]
Chlorophyll vegetation index (CVI)	Rn2 × Rr/Rg^2^	490:570, 640:760, 780:1400		[66]
Chlorophyll indexRedEdge (CIrededge)	NIRrededge−1	690:730, 780:1400		[67]
Chlorophyll index green (CIgreen)	NIRGreen−1	490:570, 780:1400		[67]
Leaf chlorophyll index (LCI)	(850) – (710) (850) + (680)	680, 710, 850		[67]

NIR, near-infrared; MIR, mid-infrared. Rn, Rr, Rb, Rg, and Rre represent the reflectance values for the following spectral bands: near-infrared (NIR) (~800–900 nm), red (~600–700 nm), blue (~400–500 nm), green (~500–600 nm), and red edge (~700–750 nm), respectively. The red edge band is characterized by a sharp rise in reflectance from the red to the near-infrared regions.

**Table 2:** Research on addressing the adverse effect of abiotic and biotic stresses in crops through the application of various machine learning and deep learning techniques.

Crops	Stresses	Growth Conditions	Spectral Range (nm)	Data Analysis	Sensing Modality	Countries Performed	References
Cotton (*Gossypium hirsutum*)	Water stress	Field	490–900, 7500–13,500	Mapping and correlation	Thermal imaging	China	[93]
	Mealybug		350–2500	Linear classification analysis and multivariable logistic regression	Spectro-radiometry	India	[94]
Rice (*Oryza sativa*)	Salinity	Field	380–760	Smoothing spline curves techniques and gene data analysis	Red–green–blue (RGB) imaging	Australia	[95]
		Greenhouse	400–500	Hierarchical grouping, Pearson’s correlation assessment, non-linear mixed effects model	Fluorescence imaging	USA	[96]
	Blast, false smut, brown spot, bakanae disease	Field	380–760	Deep learning	RGB imaging	China	[97]
Wheat (*Triticum* spp.)	Water stress	Landsat data	841–1652	Indices and regression	Multispectral imaging	India	[98]
Barley (*Hordeum vulgare*)	Drought	Field	430–890	Support vector machine	Hyperspectral imaging	Germany	[99]
		Greenhouse	400–900	Simplex volume maximization			[100]
	Powdery mildew, rust		400–2500	Simplex volume maximization		Germany	[101]
Maize (*Zea mays*)	Water stress	Field	475–840	Correlations among indices	Non-imaging	China	[102]
	Northern leaf blight		380–760	Deep learning	RGB imaging	USA	[103]
	Weeds		380–760	Support vector machine for classifying plants based on spectral features		Spain	[104]
Chickpea (*Cicer arietinum*)	Salinity	Greenhouse	380–760	Partial least squares and correlation analysis	RGB imaging	Australia	[105]
Soybean (*Glycine max*)	Drought	Greenhouse	400–780	Partial least squares regression	Hyperspectral fluorescence imaging	Korea	[105]

## Data Availability

All data generated or analyzed during this study are included in this published article.
